# How reliable and valid is the teacher version of the Strengths and Difficulties Questionnaire in primary school children?

**DOI:** 10.1371/journal.pone.0176605

**Published:** 2017-04-28

**Authors:** Meta van den Heuvel, Danielle E. M. C. Jansen, Roy E. Stewart, Bouwien C. M. Smits-Engelsman, Sijmen A. Reijneveld, Boudien C. T. Flapper

**Affiliations:** 1 Department of Pediatrics, University Medical Center Groningen, Beatrix Children’s Hospital, University of Groningen, Groningen, the Netherlands; 2 Department of Health Sciences, University Medical Center Groningen, University of Groningen, Groningen, the Netherlands; 3 Faculty of Kinesiology and Rehabilitation Sciences, KU Leuven, Belgium; TNO, NETHERLANDS

## Abstract

**Introduction:**

The Strengths and Difficulties Questionnaire (SDQ) is validated for parents, but not yet for teachers in a broad age range of children. We conducted a cross-sectional study with 4–10 years old school children to investigate if the SDQ-T can be used instead of the validated but lengthy Teacher’s Report Form (TRF) to acquire information about emotional and behavioral problems in the school community.

**Methods:**

Teachers of 453 children from primary schools were approached. Teachers of 394 children (response rate 86.9%) with a mean age of 7.1 years filled in the SDQ-T (n = 387), the TRF (n = 349) or both (n = 342). We assessed reliability by calculating internal consistency and concurrent validity (using correlation coefficients, sensitivity, specificity) of the SDQ-T compared with the TRF.

**Results:**

Internal consistency of the SDQ-T Total Difficulties Score (SDQ-T TDS; Cronbach α = 0.80), hyperactivity/ inattention- (α = 0.86) and prosocial behavior (α = 0.81) was very good. Concurrent validity demonstrated a strong correlation of all subscales of the SDQ-T with the corresponding scale on the TRF (range 0.54–0.73), except for peer problems (0.46). Using a SDQ-T TDS cut-off score > 14, the SDQ-T had a good sensitivity (90%) and specificity (94%).

**Discussion:**

The good reliability, validity and brevity of the SDQ-T make it an easily applicable questionnaire for obtaining information about emotional and behavioral problems from teachers in primary school children.

## Introduction

Mental health problems affect 10–20% of children and adolescents worldwide, and may strongly influence their functioning [1]. Mental health disorders are currently the leading causes of disability in children [2]. Accumulating evidence shows that young children with mental health problems are at risk for a range of negative outcomes. In adulthood these include psychiatric disorders, poor academic achievement and lower socio-economic status [2–4]. Prevention of these poor outcomes might be possible with early recognition and prompt mental health treatment [5].

For clinicians it is important to have multiple informants report on emotional and behavioral problems in children because these problems might be highly situational [6–8]. For example, children can be hyperactive in the school environment, because of the interaction with their classmates, but not display this behavior in the home environment in a one-on-one situation. Furthermore, for most DSM-V mental health diagnoses in children both parental and teacher information is required, as symptoms must be present in two or more settings (e.g. at home and school) [9]. Identifying the specific context in which children display emotional and behavioral problems and the impact of these problems on school functioning, may also facilitate treatment of these problems [10]. Teachers are good informants of children’s behavior because they see children on a daily basis in the school environment and have the opportunity to compare the behavior of children of similar age every day [6].

A reliable and valid questionnaire, the Teacher’s Report Form (TRF), is available for teachers to assess the extent of a child’s emotional and behavioral problems [11]. The TRF is the teacher version of the Achenbach Child Behavior Checklist (CBCL), with the same questions worded differently for teachers. Both questionnaires are often regarded as gold standards among broadband behavior rating scales [12–14]. For routine use, a major disadvantage of the TRF is its length, as it includes 113 items. Time and administrative burdens are reported to be important barriers for identifying mental health problems in the school environment [15]. The use of a short, inexpensive, easy accessible questionnaire could facilitate obtaining information from teachers [12].

The parent version of the Strengths and Difficulties Questionnaire (SDQ-P) has quickly become one of the most utilized screening instruments because of its brevity and its ability to measure both problem behavior and competencies [16–18]. The SDQ includes only 25 items, is freely available and translated in many languages (http://www.sdqinfo.org). The parent- and adolescent-report versions of the SDQ have been shown to have good psychometric properties [7,17,19–24]. In contrast, the validity of the teacher version of the SDQ (SDQ-T) has not been investigated in a broad age range of school children [7,25]. Although a recent review reported the strong psychometric properties of the SDQ-T, in most studies the SDQ-T results have not been compared with a gold standard for teachers, but with a gold standard for parents (e.g. Child Behavior Checklist or the Conner’s Parent Symptom Questionnaire) [20,23,26]. Studies that did compare the SDQ-T with the TRF have some limitations; they studied a specific age range (children age 5–6 years only) and another study used the TRF-reference data of another population than the one studied [27–29].

Clinicians are increasingly held accountable to assess mental health problems in their practice and are mandated to collaborate with parents and teachers on these problems [30]. It would be ideal for them to use the same, short, questionnaire for both parents and teachers to express their mental health concerns. The aim of our study was therefore to examine the reliability and validity of the SDQ-T with the TRF as a gold standard, in 4–10 years old children in primary school children.

## Methods

### Participants and procedure

We obtained data using a two-step procedure. In the first step all directors of 70 primary schools in the middle and eastern regions of the Netherlands were contacted and asked if they were interested to participate in the study. Seventeen schools consented to have teachers participate in the study (24.3%). Reasons for schools not to participate were time constraints or participation in other studies.

**Fig 1:**
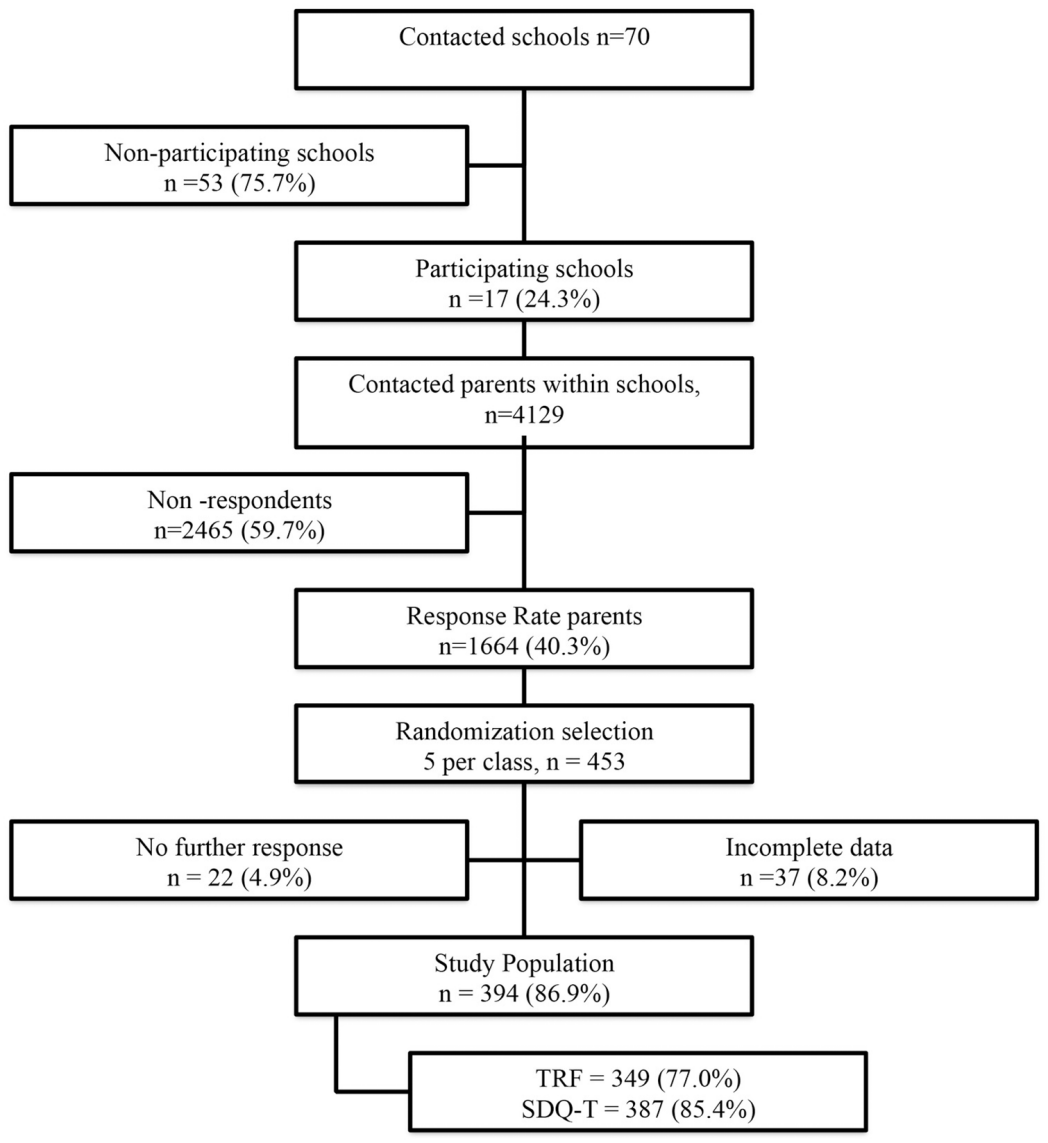
Flow chart for data collection.

In the second step, information about the study was sent to all parents (n = 4129), and 1664 parents signed informed consent (response rate 40.3%). When parents gave consent to participate in our study, they gave consent that teachers could provide information about their child to the research team. A separate consent of teachers was not obtained. A confirmation letter was sent to participating parents and teachers including the questionnaires. Since the TRF is a long questionnaire and time consuming for teachers to fill in, only five children per class were randomly chosen to participate in our study. The parents and teachers filled out the questionnaires on their own. Teachers knew their children for at least two months at the moment of filling out their questionnaires. We included 453 children. Of these, 22 (4.9%) parents did not bring back the questionnaires (“no further response”, Fig 1) and 37 (8.2) had incomplete data.

We obtained data on child and family background characteristics; i.e. age, gender, ethnic background (country of origin of parents) and educational level (highest completed grade) of parents. Parents completed the SDQ-P on 352 (response 77.7%) children. Teachers of 394 children (response 86.9%) filled in the SDQ-T (n = 387), the TRF (n = 349) or both questionnaires (n = 342). Fig 1 describes the data collection process. This study was part of a study examining emotional and behavioral problems by teachers in children with developmental coordination disorder and details of this study have been described previously [12]. This study was approved by the ethics committee of the Radboud University Nijmegen, in the Netherlands.

### Measurements

#### Strengths and Difficulties Questionnaire

The SDQ consists of 25 items subdivided into four difficulties scales, emotional symptoms, conduct problems, inattention-hyperactivity, peer problems, and a separate fifth strength scale, prosocial behavior [18]. All subscales had five questions each. An impact supplement inquired further about the existence, chronicity, and distress of problems, social- and learning impairment, and burden to others, these items can be summed to generate an “impact score” [18]. Each item was scored on a 3-point scale with 0 = ‘not true’, 1 = ‘somewhat true’ and 2 = ‘certainly true’. An example of a question in the inattention-hyperactivity subscale is: “Restless, overactive, cannot stay still for long”. We used the Dutch version of the SDQ 4–16 years for both parents and teachers, including the impact supplement. The SDQ was previously translated and validated for the Dutch setting by van Widenfelt et al. [23]. The SDQ Total Difficulties Score (TDS) was calculated by aggregating the scores for the emotional symptoms, conduct problems, hyperactivity-inattention, and peer problems subscales (range 0–40). We dichotomized the SDQ-T TDS according to Goodman’s recommendation to define the highest 10% in a clinical range (p90) [16]. In the present study population the clinical cut-off score for the SDQ-T TDS was found to be 14. Lower cut-offs may help to reduce rates of false-negatives in routine care. We therefore also calculated a second cut-off point with a lower threshold; children who scored at the 80th percentile or higher (p80).

#### Teacher’s report form

The TRF belongs to the Achenbach System of Empirically Based Assessment (ASEBA) instruments [11]. It uses an empirical quantitative approach to assess psychopathology in children. The TRF has 113 items across eight syndrome scales (Anxious/Depressed, Withdrawn/Depressed, Somatic Complaints, Social Problems, Thought Problems, Attention Problems, Rule-breaking Behavior and Aggressive Behavior) and two broadband scales (internalizing and externalizing problems) [11]. The psychometric properties of these scales have been extensively reported; the average internal consistencies were substantial for all scales; with a Cronbach’s alpha of 0.96 for the Total Problem scale and a Cronbach’s alpha of 0.92 for both the internalizing and the externalizing problems scales respectively) [31]. We used the validated Dutch version of the TRF [32]. Teachers have to indicate on 3-point scales the extent to which each item applies: 0 = ‘not’, 1 = ‘sometimes’, or 2 = ‘often’. An example question of the Attention Problems scale is “Can’t concentrate, can’t pay attention for long”. We computed the scores on the syndrome- and broadband scales, and the total problems scale. The total problem scale (TRF TPS) was dichotomized according to the Dutch cut-off score for the subclinical and clinical range [32].

### Analysis

All analyses were performed with IBM SPSS 20. First we described the background characteristics of our study population. Second, we examined the reliability, i.e. the extent to which items produce similar scores, of the SDQ-T. We therefore examined the inter-rater agreement of the SDQ-T with the SDQ-P and the scale structure of the SDQ-T by calculating internal consistencies. Inter-rater reliability was assessed with Pearson correlations for all children that had a completed parent and teacher SDQ (n = 344). We used as a benchmark the meta-analytic mean of inter-rater agreement between parents and teachers (r = 0.27) reported by Achenbach [33]. Standardized Cronbach’s alpha coefficients (α) were computed for the SDQ scales (emotional symptoms, conduct problems, hyperactivity / inattention, peer problems, prosocial behavior) impact score and total difficulties score. Cronbach’s α = 0.70 and below are generally considered as low, values between α = 0.70 and α = 0.80 as acceptable, and values above α = 0.80 as good [34].

Third, we assessed the concurrent validity, by determining the degree to which SDQ-T outcomes concurred with those on the TRF. As described in other studies, we expected correlations between the SDQ scales and the TRF scales that rated similar problems. [7,27–29] The emotional subscale of the SDQ-T was expected to have the highest correlation with the Internalizing broadband scale of the TRF. The conduct problem scale of the SDQ-T was expected to have the highest correlation with the Externalizing broadband scale of the TRF. The hyperactivity / inattention subscale of the SDQ-T was expected to have the highest correlation with the Attention Problem scale of the TRF. The peer problem subscale of the SDQ-T was expected to have the highest correlation with the Social Problem scale of the TRF. The SDQ-T prosocial subscale, which includes questions about strengths, was thought to have a strong negative correlation with the Total Problems scale of the TRF. Because of the non-normal distribution of both the SDQ-T and TRF concurrent validity was assessed using Spearman’s correlation of the SDQ-T with the TRF. Correlations below 0.30 are considered as small, correlations between 0.30 and 0.50 as medium, and correlations above 0.50 as strong [34].

We computed sensitivity (the proportion of children with emotional and behavioral problems according to the TRF TPS who are identified with the SDQ-T TDS), specificity (the proportion of children without emotional and behavioral problems according to the TRF TPS who are identified as without problems with the SDQ-T TDS) and positive predictive value (the probability that children with a clinical SDQ-TDS have emotional and behavioral problems on the TRF-TPS). We further calculated the Area Under the Receiver Operator characteristic Curve (AUC) that integrates sensitivity and specificity across the various cut-offs of the dichotomized SDQ-T TDS. A value of the AUC of 1 reflects perfect accuracy of the SDQ-T to discriminate between children with- and without emotional and behavioral problems, and a value of 0.5 reflects the absence of capacity to discriminate.

## Results

### Background characteristics

The characteristics of the study population are presented in Table 1. The study population consisted of 202 boys (51.3%) and 192 girls (48.7%), with a mean age of 7.1 years (range 4.0–10.8 years). Most parents had medium (secondary vocational education) or higher educational (university or higher vocational education) level (Table 1). The SDQ-T mean scores and the clinical cut-off scores (p90) scores and p80 scores in the total sample and stratified by gender and age are presented in Table 2.

**Table 1:** Social and demographic background of study population.

	Number (%)
*Gender*	
Boys	202 (51.3)
Girls	192 (48.7)
*Age of the child*	
4–5 year	126 (32.0)
6–7 years	124 (31.5)
8–10 years	144 (35.6)
*Ethnic background*	
Dutch	373 (94.7)
Non-Dutch	5 (1.4)
*Education Father*	
Lower education	79 (20.0)
Medium education	140 (35.5)
Higher education	141 (35.8)
*Education Mother*	
Lower education	73 (18.5)
Medium education	179 (45.4)
Higher education	113 (28.7)

Missing data: ethnic background n = 16, education father n = 34, education mother n = 29. Lower education: no education, primary education or pre-vocational education; Medium education: secondary vocational education; Higher education; university or higher vocational education.

**Table 2:** Mean scores, standard deviations (SD) and clinical cut-off scores (p90* & p80) for the teacher SDQ.

	Complete N = 387					Boys N = 199		Girls N = 188		4–5 years N = 122		6–7 years N = 124		8–10 years N = 141	
	Mean (SD)	P90 score	Percentage children ≥ P90	P80 score	Percentage children ≥ P80	Mean (SD)	P90	Mean (SD)	P90	Mean (SD)	P90	Mean (SD)	P90	Mean (SD)	P90
Emotional Symptoms	1.3 (1.9)	4	7.5	2	19.6	1.1 (1.6)	4	1.5 (2.1)	4	1.2 (1.7)	4	1.2 (1.9)	4	1.4 (1.9)	4
Conduct Problems	0.7 (1.3)	2	9.0	1	21.4	1.0 (1.5)	3	0.5 (1.1)	2	0.9 (1.6)	3	0.6 (1.1)	2	0.7 (1.3)	2
Hyperactivity / Inattention Problems	2.7 (2.8)	7	9.6	4	24.3	3.5 (3.0)	8	1.8 (2.3)	5	2.9 (2.9)	8	2.8 (2.8)	7	2.5 (2.7)	8
Peer Problems	1.2 (1.6)	4	5.4	2	17.6	1.4 (1.7)	4	1.0 (1.4)	3	1.2 (1.6)	3	1.1 (1.5)	4	1.3 (1.7)	4
Prosocial Behavior	8.0 (2.3)	5**	17.1**	6**	23.5**	7.2 (2.4)	10	8.8 (1.8)	10	7.5 (2.5)	10	8.3 (2.1)	10	8.2 (2.0)	10
Total Difficulties Score	5.9 (5.2)	14	9.3	10	18.9	7.1 (5.5)	15	4.7 (4.6)	11	6.2 (5.7)	15	5.7 (4.8)	13	5.8 (5.0)	15

*p90 score = 90th percentile score recommended for clinical use [15]

**For the prosocial scale we have used the p10 score and p20 = that means children who have score ≤ 10 percent and ≤ 20 percent respectively on the prosocial domain

### Reliability and concurrent validity of the SDQ-T

In Table 3 the results of the inter-rater agreement of the SDQ-T with the SDQ-P are presented. The correlations varied between 0.27 and 0.50. All subscales had a high correlation except the conduct problem subscale (0.27) and the prosocial behavior subscale (0.28).

**Table 3:** Inter-rater agreement of the SDQ-T with the SDQ-P.

SDQ subscales	
Emotional Symptoms	0.45
Conduct Problems	0.27
Hyperactivity / Inattention	0.50
Peer Problems	0.40
Prosocial Behavior	0.28
Total Difficulties Score	0.43
Impact Score	0.35

All correlations are significant at the 0.01 level. n = 344.

In Table 4 the internal consistency of the SDQ-T at the different age ranges are presented. The prosocial- and total difficulties scale of the SDQ-T had good Cronbach’s alphas at all ages (between 0.75–0.83). The hyperactivity / inattention scale of the SDQ-T had the highest Cronbach’s alpha at all age ranges (≥ 0.84). The Cronbach’s alpha was low for conduct problems at age 6–7 (0.44) and peer problems (0.52) at age 4–5 years.

**Table 4:** Reliability and concurrent validity of the teacher SDQ at different ages.

	Complete		4–5 years		6–7 years		8–10 years	
	*n = 387*	*n = 342*	*n = 122*	*n = 107*	*n = 124*	*n = 110*	*n = 141*	*n = 125*
SDQ-T subscales	Cronbach α	Correlation coefficient	Cronbach α	Correlation coefficient	Cronbach α	Correlation coefficient	Cronbach α	Correlation coefficient
Emotional Symptoms	0.73	0.54	0.62	0.64	0.80	0.53	0.75	0.46
Conduct Problems	0.64	0.57	0.70	0.69	0.44	0.54	0.64	0.51
Hyperactivity / Inattention	0.86	0.67	0.88	0.72	0.86	0.65	0.84	0.64
Peer Problems	0.60	0.46	0.52	0.36	0.64	0.52	0.65	0.51
Prosocial Behavior	0.81	-0.43	0.84	-0.57	0.82	-0.34	0.75	-0.38
Total Difficulties Score	0.80	0.73	0.83	0.82	0.77	0.75	0.79	0.63

All correlations were significant at the 0.01 level (Spearman’s correlation coefficient). The emotional subscale of the SDQ-T was correlated with the Internalizing broadband scale of the TRF. The conduct problem scale of the SDQ-T was correlated with the Externalizing broadband scale of the TRF. The hyperactivity / inattention subscale of the SDQ-T was correlated with the Attention Problem scale of the TRF. The peer problem subscale of the SDQ-T was expected to have the highest correlation with the Social Problem scale of the TRF. The SDQ-T prosocial subscale, which includes questions about strengths, was thought to have a strong negative correlation with the Total Problems scale of the TRF.

Table 4 also demonstrates the concurrent validity in all age ranges. We identified a strong (> 0.5) and significant correlation of all subscales of the SDQ-T with the corresponding scale on the TRF in all age ranges, except for peer problems (in 4–5 year olds) and emotional problems (in 8–10 years olds). Both the peer- and emotional problems subscales had an acceptable significant correlation (0.36 and 0.46 respectively). The highest correlation was identified between the SDQ-T TDS and TRF TPS in all age ranges (>0.73), except in the 8–10 year children that had the highest correlation (0.64) between the hyperactivity/ inattention subscale and the corresponding Attention Problem scale of the TRF.

Using a SDQ-T cut-off score > 14, the SDQ-T had a good sensitivity 90% (95% confidence interval (CI) = 0.86–0.94), specificity 94% (95% CI = 0.92–0.94) and a positive predictive value (PPV) of 0.30 (95% CI = 0.26–0.34). The area under the curve (AUC) using the TRF TPS as criterion was 0.95 (95% CI = 0.89–1.00). Supporting information is available at S1 File.

## Discussion

Our study demonstrated that the SDQ-T is a reliable and valid instrument for identifying emotional and behavioral problems by teachers in a broad age range of school children. The results of this study contribute to the literature about the validity of the SDQ-T in 4–10 year old children. The good psychometric properties and brevity of the SDQ-T make it an easily applicable alternative for the TRF to obtain information about emotional and behavioral problems from teachers of primary school children. Teachers can make an important contribution to the identification of emotional and behavioral problems in school children, as previously demonstrated in several studies [6,8,12].

Our findings on the reliability revealed a very good internal consistency of the SDQ-T TDS (α = 0.77–0.83) and of the subscales hyperactivity / inattention (α = 0.84–0.88). A low reliability of some subscales of the SDQ-T in our study (conduct problems (α = 0.44), peer problems (α = 0.52) has been reported in two other studies and in studies of the parent version of the SDQ (SDQ-P) [7,27,28]. Theunissen *et*. *al*. (2013) concluded in their study of SDQ-P in preschool children (3–4 years) that the low internal consistency of the SDQ subscales does not justify the use of these subscales to decide on a specific need of individual children for further attention regarding these problems [14]. In addition, a study by Mieloo et al. that examined the teacher SDQ in multi-ethnic 5–6 year old children in the Netherlands also identified differences in reliability of the subscales between different ethnic groups [27]. Both Theunissen and Mieloo suggested therefore using only the Total Difficulties Score of the SDQ for screening purposes; our study aligns with this recommendation [14,27]. However, an exception could possibly be made for the hyperactivity / inattention problem scale of the SDQ-T; this subscale demonstrated both the highest reliability (Cronbach’s alpha 0.88) and highest validity (Spearman’s correlation coefficient 0.72) in our study. The hyperactivity / inattention problem scale of the SDQ-T had also a very good reliability in a multi-ethnic population in the Netherlands; with Cronbach’s alphas ranging from 0.83 in Moroccan children to 0.85 in Antillean/Aruban 5–6 year old children [29]. These findings are consistent with the study of Posserud et al (2013) that demonstrated high sensitivity of the SDQ for Attention Deficit Hyperactivity Disorder [35].

Concurrent validity of all subscales of the SDQ-T with the corresponding scale on the TRF (range 0.51–0.82) was good. Our correlation results were comparable with the results found by the study of Mieloo et al in 5-year old children (correlation range: 0.43–0.76) [27]. In our study the peer-problem subscale had the lowest correlation with the TRF social problem scale. This relatively low correlation, was also reported in the study of Mieloo (2012) and Van Leeuwen (2006) [27,28]. An explanation might be that teachers are considered outsiders to the peer group of children and use an adult perspective to interpret children’s social interactions, which decreases the degree of consistency across various scales [7].

The sensitivity and specificity of the SDQ-T are slightly higher than reported for the SDQ-P in other studies [14,36]. In our study the cut-off score of the SDQ-T TDS was higher than the SDQ-P TDS cut-off score measured in Dutch parents [36]. This was also reported in a large Danish study [22]. One explanation could be that teachers may be influenced by some sort of “halo-effect” which means that children exhibiting problem behavior in one area are more likely to be rated as problematic in other areas as well, due to the impact of one class of behavior on the perception of another one [22,37]. The p90 scores for the teacher report of the SDQ in the 5-year old children in the study of Mieloo et al were remarkably lower than the 4–5 year old p90 scores in our study (p90 score SDQ-T TDS 11 versus p90 score SDQ-T TDS 15 respectively) [27]. One reason for this difference could be that we included also 4-year-old children in our analysis in this age group. The clinical cut-off point for scoring the SDQ-T TDS in 2–4 years old children in United Kingdom was also 15 [38].

Goodman’s recommendation to define the cut-off point to the highest 10% in a clinical range was based on the estimation of emotional and behavior problems in the UK population [16]. In the Netherlands, 11% of children (3–18 years) have been estimated to have externalizing behavior problems and 8% internalizing problems according to parental ratings on mental health questionnaires [39,40]. Therefore we felt justified using his 10% cut-off recommendation in our population. However, the use of other cut-offs may be justified to reduce the rate of false negatives in routine care [41].

### Strengths and limitations

The current study is, to our knowledge, the first to assess the concurrent validity of the SDQ-T compared to the TRF as a gold standard in a large community sample of 4–10 year old school children. This study did not assess construct validity of the teacher version of the SDQ since multiple other studies already identified evidence for a five-factor model of the SDQ-T [7,27–29]. A limitation is the relatively low response rate for informed consent by schools and parents. No data was collected to objectively measure if the characteristics of schools that refused to participate in our study were in any way different than the characteristics of schools that chose to participate in our study. A second limitation is that children from immigrant origin and parents with lower vocational education were underrepresented in this study. This may have led to an underestimation of the prevalence of clinical SDQ and TRF scores but it is unlikely that this has significantly influenced the correlation of the SDQ-T with the TRF [23]. A third limitation is the possible clustering of student evaluations by classroom and thus by teacher. However, each teacher assessed only five children, largely reducing the potential influence of this. A final limitation is that although the TRF is one of the best instruments available, it cannot be regarded as the ultimate gold standard, because that position is reserved for clinical diagnosis [13]. Because of complexity and high costs, structured clinical interviews such as the Diagnostic Interview Schedule for Children were not used as criterion [42].

## Implications

The SDQ-T is a reliable and valid instrument for identifying emotional and behavioral problems in primary school children. The brevity of the SDQ-T makes it an easily applicable questionnaire for obtaining information about emotional and behavioral problems on primary school children from teachers. Obtaining teacher’s SDQ ratings in addition to parental information is valuable for clinicians because this provides data on emotional and behavioral problems in children in a second setting, and can be of help in the management of mental health problems in these children [10,43]. The SDQ-T TDS may highly add to the identification of emotional and behavioral problems in the school setting in these children.

## Supporting information

S1 File SDQ study data file. (XLSX)

## Abbreviations

SDQ: Strengths and Difficulties Questionnaire
SDQ-T: Teacher version Strengths and Difficulties Questionnaire
SDQ TDS: SDQ Total Difficulties Score
TRF: Teacher’s Report Form
TRF TPS: TRF Total Problem Scale
